# Use of dupilumab for recalcitrant bullous pemphigoid: A case report

**DOI:** 10.1177/2050313X241274855

**Published:** 2024-08-22

**Authors:** Jillian Lamb, Kerri Purdy, Ashley Sutherland

**Affiliations:** Division of Clinical Dermatology and Cutaneous Science, Department of Medicine, Dalhousie University, Halifax, NS, Canada

**Keywords:** Bullous pemphigoid, dupilumab, biologics, bullous disease

## Abstract

Bullous pemphigoid is an autoimmune blistering disease affecting the dermo-epidermal junction, most commonly seen in older patients. First-line treatment includes systemic, topical corticosteroids and/or steroid-sparing immunosuppressants. Treatment with these medications may be limited by their safety profile. Dupilumab is a humanized monoclonal antibody targeting interleukin-4 and interleukin-13 cytokines currently indicated for moderate-to-severe atopic dermatitis, severe asthma, chronic rhinosinusitis with nasal polyposis, and moderate-to-severe prurigo nodularis. We report a case of a patient with recalcitrant bullous pemphigoid effectively treated with dupilumab.

## Introduction

Bullous pemphigoid (BP) occurs most commonly in patients over age 50 and is an autoimmune bullous skin disease. It is characterized by tense subepidermal blisters on an erythematous base that are intensely pruritic.^1^ Systemic corticosteroids and immunosuppressants are first-line medications for treating BP, relying on non-specific inflammation suppression and antibody production.^2^ Use of these treatments is limited by their adverse effect profiles and relapse rates after cessation. Current literature demonstrates evidence for biologics as a novel option for treating BP.^3–6^ We present a case of a 62-year-old female with severe recalcitrant BP successfully treated with dupilumab.

## Case

A white 62-year-old female presented to the dermatology clinic in March 2021 with a 1-month history of acute widespread pruritic erythema with numerous tense bullae on the medial thighs, lateral upper arms, and chest. She had a history of rheumatoid arthritis. Based on clinical presentation, she was diagnosed with BP, which was subsequently confirmed with skin biopsies for histology and direct immunofluorescence. She was started on prednisone 40 mg once daily for 3 weeks, along with doxycycline 200 mg once daily and niacinamide three times a day. The disease progressed despite increasing the prednisone dose to 100 mg once daily. She was admitted to hospital in April 2021 for severe, treatment-resistant BP. During the 3-week admission, she received solu-medrol 125 mg intravenous (IV) IV 3×, intravenous immunoglobluin (IVIG) 2 g/kg and one dose of rituximab 1000 mg IV. She was also diagnosed with hypertension and monoclonal gammopathy of undetermined significance and underwent a bone marrow biopsy to rule out myeloma. She continued to develop new bullae in friction areas; however, she was discharged home in stable condition with outpatient wound care.

After discharge, she received another dose of rituximab 1000 mg IV 14 days after the initial dose, in combination with a tapering dose of prednisone. In June 2021, she was admitted to hospital for methicillin-resistant *Staphylococcus aureus* (MRSA) bacteremia presumed to be secondary to an infection at the bone marrow biopsy site During admission, she experienced a BP flare and was started on prednisone 70 mg once daily. She was discharged on vancomycin and a tapering dose of prednisone. In November 2021, she developed more pustules that were positive for MRSA and was treated with clindamycin for 2 weeks before starting dapsone 50 mg once daily. In February 2022, she had ongoing widespread blister formation; prednisone was reintroduced at 20 mg once daily with a tapering dose.

**Figure 1.**
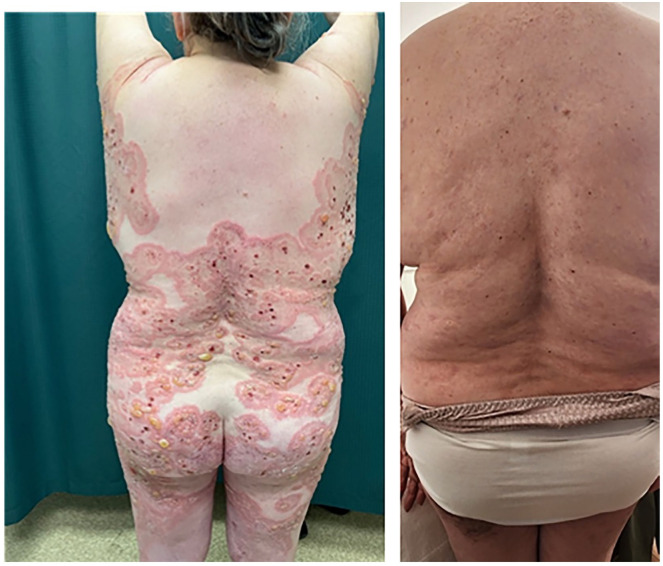
Back and dorsal aspect of proximal lower extremity. Left: Patient 28 months into refractory disease course. Right: Patient 38 weeks after initial loading dose of dupilumab.

**Figure 2.**
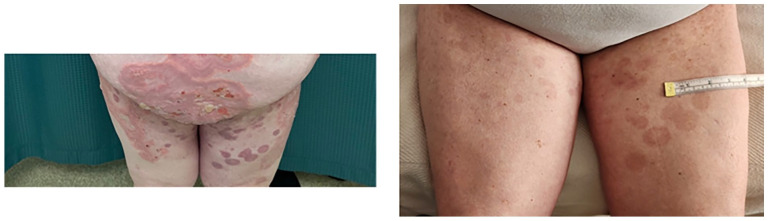
Ventral aspect of the lower extremities. Left: Patient 28 months into refractory disease course. Right: Patient 38 weeks after initial loading dose of dupilumab.

**Figure 3.**
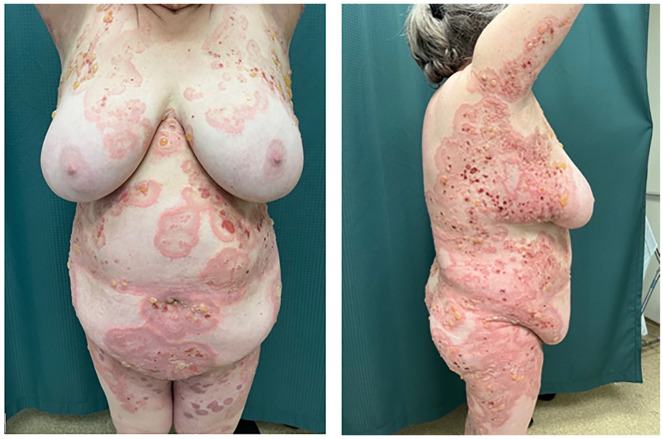
Ventral and right side of the body. Patient 28 months into refractory disease course.

**Figure 4.**
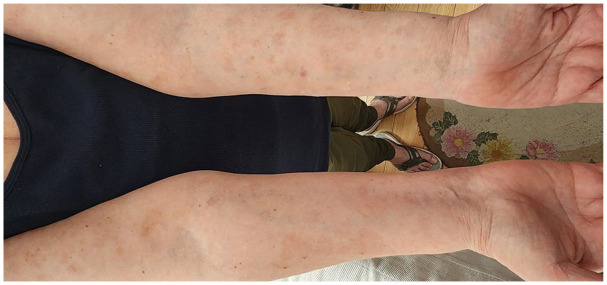
Volar aspect of distal upper extremities. Patient after 38 weeks after the initial loading dose of dupilumab.

The patient was lost to follow-up for 16 months before self-referring to the clinic with a new onset of blisters in July 2023. She had discontinued dapsone and prednisone with no active lesions until 6 weeks prior to self-referral. She denied any triggering illness but endorsed she had recently been under significant stress. On physical examination, she had urticarial plaques and tense bullae on the inner aspect of her arms bilaterally, inner thighs, trunk, and back covering approximately 70% of her body surface area (Figures 1–3). She was started on prednisone 50 mg once daily for 2 weeks, with minimal effect. Due to her prior MRSA abscess and past failure with rituximab treatment, there was reluctance to prescribe rituximab again. On August 3, 2023, she received an initial loading of dupilumab 600 mg subcutaneous injection and continued prednisone 70 mg once daily for 10 days with a slow taper. A second dose of dupilumab 600 mg subcutaneously was administered 2 weeks later, she experienced rapid improvement with clearance of the urticated plaques and no new blister formation. She was educated on self-injection administration and continued dupilumab 300 mg injections every 2 weeks. By November 9, 2023, she had tapered off prednisone with no new blister presentation. In follow-up in February and April 2024, several well-circumcised round patches of post-inflammatory hyperpigmentation scattered across her entire body and a few erythematous papules were observed on her arms (Figures 1–4). She has tolerated dupilumab well with no reports of relapse or adverse events.

## Discussion

BP is characterized by autoantibodies at the subepidermal junction against hemidesmosome proteins BP antigen 180 (*BP180*) and BP antigen 230 (*BP230*). Increased Th-2 cytokine activity, upregulation of interleukin (IL)-4 and IL-13 and eosinophil activity contribute to pruritis and bullae formation.^7,8^ First-line treatment continues to be oral corticosteroids, which are limited by long-term adverse effect profile and low efficacy for severe cases,^1^ as observed in our case. Patients with resistant disease rely on continuous corticosteroid therapy or steroid-sparing treatment (e.g., immunomodulatory agents-IVIG, dapsone, nicotinamide or suppressive agents-methotrexate, cyclosporine, and azathioprine).^8^ New research demonstrates effectiveness of certain biologics for treating autoimmune diseases, including BP.^1,3,9^ Initially, this patient was treated with rituximab unsuccessfully, corresponding with other studies that have trialed rituximab for recalcitrant BP.^7,1,10–12^ This patient subsequently failed IVIG, doxycycline, dapsone, and niacinamide and developed recurrent MRSA infections, including MRSA bacteremia, requiring hospitalization secondary to her iatrogenic immunosuppression.

Dupilumab demonstrates improved clinical symptoms, favorable safety profile, and decreased refractory cases.^12–16^ Dupilumab blocks the type 2 inflammatory pathway, targeting IL-4, its co-receptor, and IL-13.^17^ Abdat et al.,^15^ published the first multicentric case series of dupilumab use for BP in 2018, and future studies continue to demonstrate the drug’s therapeutic benefits.^7,10,18,19^

In this case, the patient was inadequately treated with conventional therapies used in BP despite adherence to medications. Dupilumab use did not demonstrate adverse effects and has continued to exhibit a promising remission course.

## Conclusion

In conclusion, dupilumab provides a satisfactory clinical response for recalcitrant BP. Future studies must confirm the optimal dosage, adverse effect profile, and maximum benefit of dupilumab for BP.
